# In vitro synergistic activity of colistin and teicoplanin combination against multidrug-resistant *Acinetobacter spp*

**DOI:** 10.1038/s41429-022-00509-7

**Published:** 2022-01-28

**Authors:** Osama Mohamed Samy Mohamed Rady, Laila El-Attar, Amira Amine

**Affiliations:** Microbiology Department, High Institute of Public Health, Alexandria University, Alexandria, Egypt

**Keywords:** Antibiotics, Antimicrobial resistance, Preclinical research

## Abstract

Drug combinations may have a crucial role in treating infections due to multidrug resistant *Acinetobacter spp*. One suggested combination is colistin with teicoplanin. The effect of colistin on *Acinetobacter spp*. outer membrane can permit teicoplanin to its target in the cell wall. The aim of this study was to evaluate the synergistic activity of colistin and teicoplanin combination against 29 multidrug resistant isolates of *Acinetobacter spp*. The antimicrobial activity of colistin alone and in combination with teicoplanin was assessed using MIC and time–kill assays. The combination of 1 mg/l colistin and 10 mg/l teicoplanin showed in vitro synergism against all tested *Acinetobacter i*solates except one (*Acinetobacter lowffii*). The combination of 1 mg/l colistin and 10 mg/l teicoplanin was bactericidal at 6 h against 100% of *Acinetobacter baumannii* isolates with no bacterial regrowth at 24 h. The same combination was bactericidal against three out of seven non-baumannii *Acinetobacter* isolates. The increased concentration of teicoplanin (20 mg/l) was synergistic but still not bactericidal against the four remaining isolates. The combination of colistin and teicoplanin was synergistic against all tested *Acinetobacter spp* It is therefore recommended that clinical trials are conducted to clarify the therapeutic potential of the combination.

## Introduction

*Acinetobacter* species (spp.) has emerged in recent years as one of the opportunistic healthcare associated pathogens. *Acinetobacter baumannii (A. baumannii)* is the most frequently encountered spp, but other *Acinetobacter* spp. are also frequently isolated. *Acinetobacter* spp. is particularly problematic in intensive care units infections and is linked to high morbidity and mortality as well as extended hospital stays. This is coupled with the increase in infections due to multidrug-resistant (MDR) *A. baumannii* and extensively drug-resistant (XDR) and pandrug-resistant (PDR) isolates that have also emerged. MDR is defined as strains resistant to at least one agent in three or more antimicrobial categories. XDR is defined as strains resistant to at least one agent in all but two or fewer antimicrobial categories. PDR is defined as those resistant to all agents in all antimicrobial categories Traditionally, carbapenem antibiotics have been considered the final line of defense however, carbapenem resistant *A. baumannii* have disseminated worldwide [1, 2].

The emergence of strains resistant to all clinically used antibiotics has led to reliance on the polymyxins as a last resort. The International Consensus Guidelines on using the polymyxins has recommended that both colistin and polymyxin B be made available for the physician to have the flexibility to use either of them depending on the clinical situation. In vitro these antibiotics behave similarly and therefore we have used colistin in this study but expect that polymyxin B could behave similarly. Colistin remains an effective antibiotic against *A. baumannii*, however, colistin resistance in *A. baumannii* has been reported. Another problem associated with colistin is heteroresistance which raises concerns that colistin alone may lack sufficient killing activity to be used as a monotherapy. Thus, the use of combination therapy has been recommended as a potential strategy to boost bacterial killing and decrease the development of resistance in treatment of MDR *A. baumannii* infections [3]. One of the suggested combinations is colistin with the anti-gram positive antibiotics. such as the glycopeptide teicoplanin. The adjuvant permeabilizing effect of colistin on the outer membrane can allow teicoplanin to penetrate the cell and act by inhibiting cell wall synthesis in dividing organisms [2, 4].This study aimed to evaluate the synergistic activity of colistin and teicoplanin combination in vitro against *Acinetobacter spp*.

## Materials and methods

### Bacterial isolates

Twenty-nine *Acinetobacter* spp. isolates were collected from clinical microbiology laboratories and verified using MALDI-TOF MS (Autoflex, Bruker Daltonics, Germany). Fifty percent of bacterial isolates were collected from respiratory samples (30% from bronchial lavage and 20% from sputum), 26.6% from blood, 10% from pus swabs, 6.7% of each of urine and environmental samples.

Twenty-two of the identified isolates were *A. baumannii*, 4 *Acinetobacter nosocomialis (A. nosocomialis)* isolates and one of each of *Acinetobacter lwoffii* (*A. lowffii)*, *Acinetobacter junii* (*A. junii*) and *Acinetobacter haemolyticus* (*A. haemolyticus*).

### Antimicrobial susceptibility testing

Susceptibility patterns of all isolates were tested using a panel of 13 antibiotics including, piperacillin, piperacillin-tazobactam, ampicillin- sulbactam, ceftazidime, ceftriaxone, imipenem, meropenem, gentamicin, tobramycin, amikacin, doxycycline, ciprofloxacin, levofloxacin, and trimethoprim-sulphmethoxazole [5].

### Colistin MICs determination

Colitin MICs were determined using commercial kit ComASP^TM^ Colistin (Liofilchem®) for the 29 identified bacterial isolates [6]. Breakpoints were interpreted according to CLSI 2019 [5].

### Time–kill assays

Time–kill assays were performed using colistin sulfate at a concentration of 1 mg l^−1^ and teicoplanin at 10 mg l^−1^. Bactericidal activity was defined as a three-fold log reduction in cell numbers compared with the starting inoculum. Synergy was defined as a ≥2-fold log reduction in cell numbers at 24 h when compared to the most active agent used alone [7].

Bacterial isolates that the combination of colistin 1 mg l^−1^ and teicoplanin 10 mg l^−1^ was not bactericidal and/or synergistic against were tested again using the combination of colistin 1 mg l^−1^ and teicoplanin 20 mg l^−1^.

## Results and discussion

All the tested isolates were 100% resistant to ceftriaxone and ceftazidime. For the other tested antibiotics the bacterial isolates showed resistance ranging from 33.33% to 96%. According to their pattern of resistance to tested antibiotics, 15 isolates were found to be MDR (51.7%), 11 XDR (37.9%) and 3 PDR (10.3%). Five of the tested isolates showed resistance to colistin, these were 2 of each *A. baumannii* and *A. nosocomialis* and one of *Acinetobacter lowffii* (*A lowffii*) (Table 1).

**Table 1:** Distribution of the 29 bacterial isolates according to their susceptibility to colistin

Isolates	Colisitn MICs (μg ml^−1^)						
	Colistin susceptible MICs ≤2 μg ml^−1^				Colistin resistant MICs >2 μg ml^−1^		
	0.25	0.5	1	2	4	8	16
*A. baumannii* (22)	13	6	1	–	2	–	–
*A. nosocomails* (4)	2	–	–	–	–	2	–
*A. lowffii* (1)	–	–	–	–	1	–	–
*A. junii* (1)	–	–	–	1	–	–	–
*A. hemolyticus* (1)	1	–	–	–	–	–	–
Total	17	6	1	1	3	2	–
	24					5	

Colistin’s bactericidal activity against *Acinetobacter* spp. is concentration-dependent, and an average plasma concentration of 2 μg ml^−1^ colistin has been proposed as a for isolates with MICs of ≤1 μg ml^−1^. However, this is difficult to achieve clinically [8]. So, in the present study colistin was tested in concentration of 1 mg l^−1^ to make it more clinically relevant. Since colistin is already nephrotoxic, teicoplanin was selected instead of vancomycin as it has lower nephrotoxicity so the combination can be more clinically relevant. Teicoplanin optimal therapeutic plasma concentration is suggested to range from ≥10 μg ml^−1^ to ≥20 μg ml^−1^ [9], and the lower concentration of teicoplanin (10 mg l^−1^) was used in this study.

Colistin and teicoplanin combination was tested against 29 tested *Acinetobacter spp*. isolates. This combination was synergistic and bactericidal against the 22 tested *A. baumannii* isolates. Previous studies have examined synergism between colistin and teicoplanin, however a smaller number of isolates were used, and the optimum concentration of each antibiotic was not defined, as different methods and the difference in the clonality of isolates resulted in different outcomes. To our knowledge, there are no previous studies that tested the combination on non- *A. baumannii* isolates, while the present study included 7 isolates (Table 2).

**Table 2:** Colistin sensitivity and synergy testing with teicoplanin

Strain (number)	Colistin MIC mg/l	Time to kill assay		
		Colistin 1 mg l^−1^	Colistin 1 mg l^−1^ and teicoplanin 10 mg l^−1^	Colistin 1 mg l^−1^ and teicoplanin 20 mg l^−1^
*A. baumannii* (20)	0.25–1	Sensitive, regrowth after 24 h	Bactericidal at 6 h, no regrowth after 24 h	ND
*A. baumannii* (2)	4	Resistant	Bactericidal at 6 h, no regrowth after 24 h	ND
*A. nosocomialis* (2)	0.25	Sensitive, regrowth after 24 h	Bactericidal at 6 h, no regrowth after 24 h	ND
*A. nosocomialis* (2)	4	Resistant	Synergistic, no regrowth after 24 h	Synergistic no regrowth after 24 h
*A. lowffii* (1)	4	Resistant	Not synergistic, regrowth after 24 h	Synergistic no regrowth after 24 h
*A. junii* (1)	2	Resistant	Synergistic, no regrowth after 24 h	Synergistic no regrowth after 24 h
*A. haemolyticus* (1)	0.25	Sensitive, regrowth after 24 h	Bactericidal, no regrowth after 24 h	ND

*ND* not done

**Fig. 1:**
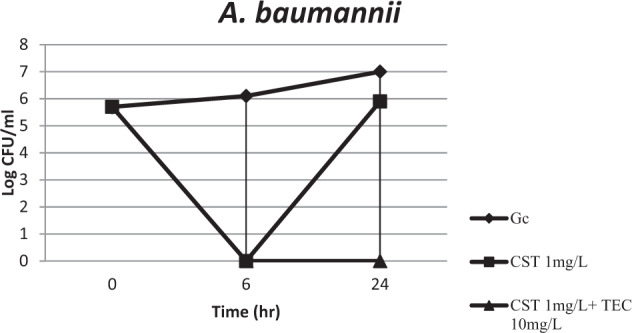
Time–kill assay performed on 20 colistin susceptible *A. baumannii* isolates in the presence of 1 mg l^−1^ colistin (CST); and 1 mg l^−1^ colistin + 10 mg l^−1^ teicoplanin (CST/TEC). Gc growth control

Wareham et al. tested the combination on five MDR colistin- susceptible isolates of *A. baumannii*, however we used a lower concentration of teicoplanin (10 mg l^−1^) [4]. The present study included only two colistin-resistant *A. baumannii* isolates where colistin-teicoplanin combination was bactericidal and synergistic against both isolates. Bae et al. [10] used higher concentrations to test the synergistic effect of colistin (2 mg l^−1^) and teicoplanin (16 mg l^−1^) against colistin-resistant *A. baumannii* isolates. However, their results depended on the method used, as synergy was higher using checkerboard methodology (45%) versus multiple-combination bactericidal test (88.88%). Bae et al. suggested that the combinations of glycopeptides and colistin may be effective regardless of its MICs, due to an adjuvant permeabilizing effect of colistin on the *A. baumannii* outer membrane (Fig. 1).

Bacterial regrowth was observed in this study. It may have several reasons in vitro including the use of sub-inhibitory concentration of antibiotics, emergence of resistant subpopulations, adherence of bacteria to the surface of the culture vessel and inactivation of the antibiotics in vitro are reasons for bacterial regrowth [11]. Moreover Owen et al. [12] observed that colistin was very active in the initial killing of colistin-susceptible strains of *A. baumannii*, even with 0.5 × MIC. However, a modest positive post-antibiotic effect of colistin was noticed at higher concentrations (≥16 × MIC), which cannot be achieved in clinical practice and there was the substantial regrowth occurring at 24 h even at colistin concentrations up to 64 × MIC.

Sanderink et al. [13] tested the efficacy of colistin–teicoplanin combinations in-vivo, the colistin-teicoplanin increased the survival of mice infected with *A. baumannii* murine model of pneumonia. Sanderink et al., results suggest the possibility of using the colistin–teicoplanin combination in certain therapeutic deadlocks.

In the last decade, growing numbers of human infections caused by the non-baumannii *Acinetobacter* even MDR isolates causing hospital acquired infections have been reported globally. Even species that have less typically been linked to human disease including *A. lwoffii*, *A. junii*, and *A. haemolyticus*, were also reported [2, 14].

Non-baumannii *Acinetobacter* spp. have been shown to be resistant to colistin more often than *A. baumannii*. Several studies have reported a high level of resistance to colistin in *A. nosocomialis* compared with *A. baumannii*, ranging from 6.5 to 45.3% [15–17]. These findings are in accordance with findings of the present study as colistin resistance rate was 9.1% (2 out of 22) in *A. baumannii* while it was 40% (2 out of 4) in *A. nosocomialis*.

In the present study, the combination of colistin 1 mg l^−1^ and teicoplanin 10 mg l^−1^ was synergistic against all non-baumannii isolates except one (*A. lowffii*), which was synergistic when teicoplanin concentration was increased to 20 mg l^−1^. In contrast to *A. baumannii*, the synergistic activity of the combination was not bactericidal against more than half of non-baumannii *Acinetobacter* isolates. Two of these isolates were *A. nosocomialis* and had a high colistin MIC value of 8 mg l^−1^.

The difference in the bactericidal activity of the combination on *A. baumannii* and non-baumannii *Acinetobacter* could be attributed to the difference among *Acinetobacter* genospecies in their antimicrobial susceptibility [18, 19] and mechanisms of resistance to antimicrobial agents [18]. Moreover, in the case of non-baumannii *Acinetobacter* spp., information regarding the mechanisms of colistin resistance remains limited [20]. Although that *A. lwoffii* is usually susceptible to colistin, the present study included *A. lwoffii* clinical isolate that was colistin resistant Since *A. lwoffii* is not commonly found in clinical practice, this offered little opportunity for investigation thus its mechanism of resistance is still not clear, but maybe due to mutation in its lipopolysaccharide component.

In conclusion, the combination of colistin and teicoplanin was very effective in examined concentrations against all tested *Acinetobacter spp*. It is therefore recommended that clinical trials are conducted to clarify the in-vivo therapeutic potential of this combination.
